# Comparison between wet and dry lab in low-fidelity simulation of mitral valve surgery

**DOI:** 10.1186/1749-8090-8-S1-P162

**Published:** 2013-09-11

**Authors:** A Hossien, I Khan, H Subhani, S Ashraf

**Affiliations:** 1 Cardiothoracic Surgery Department, Morriston Hospital, Swansea, UK

## Background

Nowadays the training in mitral valve (MV) surgery is competitive and difficult due to reasons including limited exposure, complexity of 3D structure of MV, complexity of surgical techniques used in MV repair and low rate of MV surgery in some centres. Therefore low-fidelity simulation in MV surgery becomes a necessity. We compare between the training in dry and wet lab simulation in this study.

## Methods

We performed different MV repair techniques using MV models in a dry lab and porcine hearts in wet lab. The properties of materials used in both models were tested e.g tissue handling. The possibility of 3D construction of normal and pathological MV and the ease of repair were examined. Availability, flexibility, portability, disposal of remains and cost of organizing both environments were noted. The number of procedures performed on each model was recorded.

## Results

The self 3D construction of MV is only possible in dry model, which will enhance the understanding of 3D complexity of MV components and allows creation of pathological models. The porcine heart allows performing MV repair techniques with closer resemblance to the human heart in mechanical properties. The availability, flexibility and disposal of remains are feasible in dry lab because the constructing materials are readily available and there is a lack of need of proper storage e.g. refrigeration. The dry model is portable and it can be used every time and everywhere. The number of procedures performed on the dry lab was higher than wet lab due to the nature of material which is reusable, recyclable and cost effective.

## Conclusion

The dry and wet labs are valuable tools in improving training in MV surgery. There are more advantages of using the dry model initially as low fidelity simulator than the wet model because of flexibility, availability, low cost, the possibility of self modelling of normal and pathological MV and it can be used for an unrestricted number of procedures.

